# Materia Medica and Its Practical Uses

**Published:** 1882-08

**Authors:** P. P. Wells

**Affiliations:** Brooklyn, N. Y.; Bureau of Materia Medica, etc., I. H. A.


### MATERIA MEDICA, AND ITS PRACTICAL USES.

P. P. Wells, M. D., Brooklyn, N. Y.
(*Bureau of Materia Medica, etc., I. H. A.*)

What is materia medica? What do we mean by these words? The reply is determined by our status as members of the profession of practical healing. The medicine of antiquity will answer: Materia medica is a classified catalogue of substances which have power to affect and change the functions of some organ of the human body; and the classification of those substances is based on the fact of the greater affinity of any one of them for one organ or function rather than for others. For example, a substance which acts on the stomach, provoking the rejection of its contents, is an emetic; and, beyond this fact, the practitioner does not concern himself. If the drug be Ipecacuanha, the fact that it will excite vomiting is sufficient to refer it to this class, and its manifold and important actions on other organs and functions are ignored. If the one sole use to be made of a drug were to classify it, this would be sufficient. But if the objective of the knowledge of drugs be the cure of the sick, then other powers of a specimen may be even more important than that which has been seized upon as its classifying characteristic. Thus, the curing power of Ipecacuanha has a broad range beyond that of its specific action on the gastric functions. This is of no interest to teacher or pupil, where the first and chief object is classification. It is an emetic, and that is sufficient. Other effects from its use are sometimes recognized even by these one-eyed observers; but they are readily referred to this first and most important function of the drug, as resulting from this. For example, its action on the skin is only the effect of the physical exertion necessary to the act of vomiting. Its effects on the respiratory or nervous functions—well they were only by the way affairs, to one who is so entirely intent on classification, and not worthy of especial notice; and the more so, as if seriously regarded, they would only become disturbing elements in this classification, which is so conveniently rested on this one peculiar effect upon this one organ and function. So of other drugs in this system, the one grand characteristic of which is classification. If a substance is found to evacuate the lower alimentary track of its contents rather than the stomach, it is not allowed to associate with its upper neighbors, the emetics, at all. It must go to the cathartics and there abide. So of diuretics, sialogogues, emenagogues, etc., to the end of the chapter, each drug finding its place determined by its being found to select some one organ or function rather than others. To be sure, the classifiers were often puzzled and embarrassed by the multiplied effects of their administered drugs, and oftener by mercury than by others. Its notable action on the salivary glands compelled its standing a sialogogue. But then the miserable drug was of so unruly a nature it was found to be wholly regardless of the beauties of science, especially so much of these as were bound up in this convenient system of classification. After being so placed in its true family connection, for science's sake, because it increased the flow of saliva, what right had it to go on and vomit and purge patients, inflame and destroy their bones, excite eruptions on the skin, and "cut up" in a very general and unruly way among all the other organs and functions of the body, as if it had been created only to pour contempt on honest people's efforts to bring drugs into scientific groups, each characterized by some one special defining feature.

If so to do is not very respectful to these honest and earnest men, it cannot be denied that it is characteristic of drug action. The obstinate and thoughtless agents will act out their own nature when in contact with the living human organism, and are not at all likely to regard preconceived notions of even wise and honest men when once they have the field to themselves.

It is notable in this attempt to bring drugs into classified groups, there is no apparent thought of them as specifically related to sicknesses which are to be cured by them, nor any inkling of any principle by which they might possibly be so related. It is only a question of vomiting, purging, sweating, etc., and there the individual drug is placed in its supposed appropriate group to await a call into practical use, according as the imagination of the prescriber may fancy it to be a beneficial agent in this or that malady, guided to this fancy by no shadow of a principle or reason other than this of his individual judgment, that it will *somehow or other* do his patient good. It is the rarest that any thought of a specific relationship of the drug to the sick condition to be cured, enters the mind of him who gives it in this general way. The sicknesses are few in which this rare occurrence is met. An instance of this may be found in the blind and arbitrary giving quinine for the cure of all cases of ague, and an equally blind giving of mercury for syphilis. It never seems to enter the heads of these classifiers that these drugs cure these diseases, when they do cure them, for any other or better reason than this: they cure because they cure; or to inquire whether there be a principle of more or less general character which constitutes the power of these drugs to cure these diseases. They repeat the giving of the quinine because the case is ague, and for this solely; and they are content to do this while they stolidly and loudly claim for this stupidity that it is a "*scientific*" practice of medicine, though the only semblance of a science in the whole proceeding is found in this lame attempt at classification of the drugs employed, and notwithstanding the utter absence of all principles to guide in the selection of the drugs they use.

There is a radical defect in this classified materia medica, in that it has no class characterized by especial effects on the intellectual and moral forces and functions, which leaves a large field of most important maladies wholly unprovided for. Its utmost reach in this direction is to endeavor to quell cerebral excitements by the stupefaction of narcotics, or by revulsions by means of raking irritation of the alimentary track by purgatives of more or less violence. In lower grades of excitement, this system is sometimes called on for a drug which has been arbitrarily classed as an *antispasmodic*, and then for treating mental diseases its resources are exhausted. Narcotics, purgatives, antispasmodics and counter-irritants, so-called, and *voila tout.* And yet this is the school which claims to be recognized as the possessor of all knowledge and resources in medicines, and affects to regard with contempt whoever approaches it with suggestion of larger resources or better means. Do they do this on the principle of the beggar who objects to having his rags exposed to general view?

The *intent* in the use of these classified drugs by the practitioners of this school is as utterly destitute of directness of object as its classification is of all semblance of reason. With the exception of the very few drugs which they wrongfully claim as specifics for an equal number of diseases, the school attempts its cures almost wholly by indirect, round-about means, as by revulsions, counter-irritants, etc. It has no thought of any principle of relationship between drugs and diseases by which a drug becomes the specific curative of a given sick condition, consequently, prescribing for the sick by this school is always in the dark and, therefore, always a matter more or less of guessing, and yet, in their own language, theirs is, *par excellence*, the "*regular*" school of practical medicine, "*regular*" only by reason of the unvarying *guessing* which characterizes it.

Is this all of the materia medica of the antique school of the present day? Not quite. It is a fair representation of it till its teachers and writers in modern times invaded sources of knowledge outside its own circle, gathered by the industry and intelligence of those whom these authors affect to hold in contempt and hate, and from whose labors they have not hesitated to pillage all which gives to their work even a semblance of a statement of knowledge of the specific effects of the drugs of which they treat, and in which plunder is found all which gives value to their work. While thus engaged, it is difficult which most to admire, their unquestioned good judgment in selecting their forage ground or their perfect reticence as to any, even the slightest, hint of the source from which these gatherings came, which they have so unblushingly given to the world as their own. It is said even Satan sometimes compliments truth and virtue by endeavors to imitate them, but we have never heard of his *stealing* them and giving them out as his own.

But what has the new school of healing to answer to the question, What is materia medica? It is the sum of the known powers of drugs to affect the organs and functions of the human body, changing these so that the balanced action of the life-force in these, which we call health, is disturbed and the result is disease or sickness. All substances having this power are drugs. All drugs, then, are sick-making substances. All drugs are healing agents which have the power to change these organs and functions, when made sick by other causes, to the normal balance of their life-force, *i. e.*, to health.

The new school requires, before a drug can be incorporated into the family of its materia medica, not that it be classified, but that it be given to a person or persons in whom the balance of life-force is perfect, *i. e.*, in health, and that the disturbances produced be carefully observed and a written record be made of them as to each and every organ and function, with all the conditions and circumstances in which these appeared and by which sufferings consequent on these disturbances are either aggravated, relieved or changed in their character. All this is required of each member of the family and in the utmost possible detail. This is the "*proving*" of the drug. The early provings by the author of our school and his co-laborers were, after the records of the day-books were handed in to the master, subjected to the severest scrutiny, that all mere natural sensations and facts from other causes than the drug, found in the record, by reason of want of knowledge or proper caution, might be eliminated. It was this scrutiny and elimination which has given to these early provings their practical reliability and unequaled value, while the absence of these has stamped so many of the more modern provings as of little worth and many of them as utterly without value as healing agents.

It will be seen that in the idea of a materia medica, the two schools have little that is common to the two The one accepts whatever will vomit, purge, narcotize or increase any one of the secretions of organs, and proceeds to gather these agents into groups according as they have been found to have produced these effects, and with this they were satisfied till they resorted to pillage to give to their work a greater seeming of scientific character, by incorporating the fruits of other men's labors into their own in the form of specific effects of some drugs on particular organs or functions of the body. But after this stealing they were but little better off as healers than before, as their plundered goods left them to their old indirect method of the use of drugs, having no principle or law to direct in their choice. The other, before acceptance of any drug, will have a knowledge of the effects it can produce on each and every organ and function with complete detail of the circumstances and conditions in which these have been produced, aggravated or relieved.

These differences are hardly greater than are the views taken of the objects on which the two schools propose to act by the agency of the resources of their materia medica. The one from the generalization of a few facts assumes an internal condition of his patient (calls this *pathology*), which by setting up a different internal condition, he proposes to remove in this roundabout way. If he is not very clear as to just how this is to be effected by means which, so far as he knows, have no specific relation to the malady in hand, he still looks for this result with so robust a confidence that he claims for this method, to the exclusion of all others, that it alone is to be accepted as the only "regular" and "scientific" example of practical medicine.

As opposed to this, the new school takes the perceptible facts of the case, as revealed in sensation and perverted function, which alone disclose the aberrations produced by the impress of the morbid cause, and declare to the practitioner their true nature, while, at the same time, they give to him the only sure guide in his selection of their specific curative. By the administration of this, when found, he proposes a restoration of these perverted functions and sensations, which alone constitute the "*disease*," to their normal balance of force and action, which alone is *health*, by the direct and specific action of the curative on the morbidly-affected organs and functions; and this, by means which leave other organs and functions unaffected by their presence. Thus it will be seen the new school opposes to the indirect method of the old, its own direct application of its one specific curative. It proposes thus the extinction of the perceptible abnormal facts of the case, as opposed to the imaginary internal condition of the old.

There is a difference in the new school itself, as to the form in which the curative, when found, shall be given to the sick for their healing. While the old school give crude drugs in their natural state, the new, for the most part, give them in a form to which they have been brought by artificial manipulation. The process by which this change is effected, is generally called "*potentization*."

The difference in the new school, to which we have alluded, is found in the estimation of the value of this process, and in the judgment of how far this shall be carried, in order to constitute the drug a remedy, with the greatest possible power for healing. The one party holds, that the presence of the material visible molecules of the substance of the drug is required for experience of its curative power; taking for granted that it is in these material molecules that the curing power of the drug exists; thus assuming as a fact, what they have not proved, and what, by the other side, is denied, and the denial, it is claimed, is fully sustained by their manifold and abundant clinical experiences. This side, claiming that the material molecule is the representative and embodiment of the curing power of the drug, and that the size of these, and their number in a cubic inch, have been revealed by the microscope, the micrometer, and the multiplication table; and, that these demonstrate, according to testimony of careful and competent observers, that the utmost division of material molecules is reached in the eleventh number of the centesimal series, in the process of so-called potentization, and *therefore*, all the so-called "*potencies*" above this number, can only be a continuation of the actualities of this eleventh number, whatever they may be, or be held as nonentities as healing agents to the end of the series, however extended. Admitting what this party assume as the basis of this argument, the *materiality* of the curing power, and it is not easy to escape their conclusion as to the limitation of medicinal potentiality, either at the eleventh centesimal or at some other number of the series, where vision with the microscope fails to detect the presence of material drug molecules. They affect to limit this in the solids by the use of the microscope, and in the solubles by the multiplication table and fractions of millimetres. There can be, by physical possibility, only a certain number of these molecules in the cubic inch, and, therefore, all pretense that a greater division of these, after the known limit of the possible divisibility has been passed, has resulted in a greater development of curing power in the drug, is simply an absurd impossibility. This argument is also based on an assumption that increased medicinal power is the result of increased division of material molecules; an opinion which many have held, and which many are ready to say is absolutely negatived by their own practical experiences. The argument, then, on this (which we will call the material) side, is that the curing power is the material substance of the drug. This material is capable of a reduction to a molecular state, in which each molecule has a definite size, and beyond this, possible division cannot reduce them. And as the curing power is the molecule, and is determined by the number of these brought into contact with living sick surfaces, and, as beyond a certain point this number cannot be increased, therefore, beyond that point there can be no increase of curing power by the continued process of potentization, *i. e.*, beyond this point there *is no* potentization.

The other side (which for convenience we will call the dynamic), while using the same agents, brought to their knowledge and confidence by the same provings, and using them under the guidance of the same law, start with different views of the nature of the curing power. They deny its existence in the material molecule, or that it is, as potentized, represented by that molecule. They hold that potentization has developed and set this power free from the molecule; so that, as to all practical uses of this power, it is a matter of utmost indifference whether the microscope sees or does not see the material particles of the drug, or whether a cubic inch of water contains "fifteen," or "fifty trillions" of molecules, this power being in no sense or degree detected by the instrument, or capable of expression by the multiplication table. This party accepts the limitation of the other as to the visibility of solid particles of drug matter, and as to the possible limit of particles in a cubic inch of any fluid; but, it denies that this has anything to do with limiting the development of the curing power of drugs by the process of potentization; on the contrary, it affirms that these asserted material facts prove the assumed facts, as to the nature of the curing power and the limitation of this to the possible range of the microscope, or the number of molecules in the cubic inch, to be altogether false. Indeed, they deny that the power can be detected, or measured by the microscope, or micrometer, or by the number of particles in a cubic inch, or expressed by any range of arithmetical figures. It affirms that this power which cures, is altogether independent of optical instruments and mathematics, and is in no way related to them, and therefore, can in no way be demonstrated by them, either affirmatively or negatively. So far is the development of this power from limitation to the eleventh, in the centesimal series, the limit of the microscopic detection of particles of solid drug matter, these dynamic men claim that they have found these same solid drugs curatives of sicknesses in numbers very much higher than the eleventh, when lower numbers of the same drug had failed; from which they claim a demonstration, in view of these discoveries of the materialists, that the curative power of the drug is not matter.*

If they are not mistaken as to these experiences, they remove the microscope and mathematics from the argument as to potentization. These experiences have been too numerous to be set aside by mere negation. They belong to men too numerous and too respectable to be ignored. In amount, they are equal to establishing any truth which can be established by human testimony.

It will be seen that the difference in the new school as between the materialists and dynamists, has its origin in the erroneous views of the former, as to the nature of the power in drug agents which cures. They claim for this, that it is in the matter of the drug, and on this they hang their faith and practice. Starting from a false premise (the material nature of the drug power), it could not be otherwise than that they should come to a wrong conclusion. There can be no hesitation in saying their premise (the material nature

---

* The following case is an example of this experience, and is also a speaking witness to the truth that the curing power of drugs is not material, if, as the materialists say, the matter of the drug ceases to be present after the eleventh of the centesimal series. The writer was called to a neighboring city to a consultation on the case of Mrs. W., sixty-five years of age, suffering from an attack of Asiatic cholera. It was the first case of the kind the attending physician had seen, and he called for help. After a careful examination of the case, there was no doubt as to its remedy. It was given, with temporary relief, in the 200th, 30th, 12th, 3d, and tincture, each number acting as a temporary check to the symptoms and sufferings of the patient; but these, after a short interval, returned, and the case made a downward progress in spite of them all. I saw the patient in circumstances peculiarly adapted to favor the reception of the cholera miasm, if this can be taken from one individual by another. I had an attack of the disease in a few hours, and was next day visited by the attending doctor while in my bed, when he gave me the above account of the effect of his doses, and the present state of his patient. After careful study, the same remedy was pronounced best, and, if the best would not cure, nothing would. He left, and on his way home, determined to give a dose of Fincke's 40,000, which he did; and after taking it, the patient had neither vomiting, purging nor cramps. She was perfectly cured, and her convalescence was remarkably short and without interruption; which, considering her age, was a little remarkable. This 40,000, whatever may be said of its real status in the centesimal series, was certainly *above the eleventh.*

of the drug power), is false even if judged solely by their own alleged discoveries. Indeed, these are quite sufficient to disprove this nature if taken in connection with the many thousand times repeated experiences of the dynamists. The experiments prove no drug molecules beyond the 11th. But the dynamites have seen thousands and thousands of cures result from the use of numbers much above the 11th, and, therefore, it follows, as there is no drug matter in these numbers, the power that cures is not matter. The experiments and observations of the materialists themselves demonstrate the falsity of their fundamental basis of opinion.

The dynamists, on the other hand, regard this power as an immaterial principle embodied by the Creator in the drug, capable of separation from its material association by the process of potentization, and by this, so far as now known, of an indefinite expansion and increase of energy, giving to curing agents a greater efficacy, and an interest to observing minds altogether new and unique. The materialist rejects this view and so denies himself and his patients the benefits inhering in this new energy and increased efficacy, because (and this is the only *reason* I have seen for this rejection other than the will of the objector), forsooth, the microscope can show no drug particles in numbers higher than the 11th, and, a cubic inch of a fluid of water, for example, can contain but 15 trillions of molecules; therefore, there can be no such expansion and increase of energy, as the experience of the dynamists affirm there is, this being from a standpoint of view of the materials, a physical impossibility. Against this, the dynamist opposes his cures with high, and even very high numbers, and his vastly better success in healing with these; and, he says, in view of the declarations of the materialists, that their experiments with microscope and micrometer, and their resort to mathematics prove conclusively the falsity of the material idea, and confirm their own of the dynamic nature of the curing power of the drug.* The argument so put

---

* The following experience in the practice of one of my most intelligent professional neighbors, shows that the power to *produce* disease, with all its phenomena in perfection, is present in potentized numbers, even in those which are far above the famous 11th. Miss F., 14 years of age, was given a single dose of *Variolinum* 900 centesimal as a prophylactic of *small-pox*. There were no examples of the disease in the neighborhood, nor had the patient been in any way exposed to its contagion, so far as known. After the dose, and the usual period of incubation which follows this contagion, she had the initial would seem to be final and in favor of the dynamists. There is but one escape from this apparent; and of this the materialists are not slow to avail themselves. It is the *non credo*. But this is not argument. That they do not believe cures have been and are wrought by high and higher numbers than the 11th, and in a much more perfect manner than by lower numbers, as affirmed by the dynamists, does not extinguish a single fact contained in these experiences. The patients have been and are cured all the same, and the skeptic's *non credo* has no power to prevent such cures, by such agencies given to the sick by whoever will qualify himself to gather the facts of the sickness, and give the labor needful to find by a comparison of these with the facts of the materia medica, the one drug in the recorded effects of which is found the greatest likeness to those of the disease.

*Non credo* adds to no man's knowledge. It is only an effectual bar to all advancement. It is an offspring of that egotism which refuses testimony as to facts, which self has neither observed nor experienced.

But why reject the testimony of these witnesses to cures made, under their own observation, by agents in which the microscope declares there is no drug matter? What then? The cures were made, these witnesses affirm; and, this being a fact, one of two things follows, from which there is no escape; either the curing power is not matter, or the sick sensibilities have detected matter where the microscope could not. These witnesses, who testify to these cures so made are many; their integrity is unimpeached; there is no evidence that they are not as to capacity for accurate observation fully the equals of the average of those who cry *non credo*. Then, we repeat, why reject their testimony? Is it hinted that, if honest, these witnesses are self-deceived? The great number who testify to these cures, and with such unanimity of experience, makes the idea of self-deception the greatest of absurdities. Indeed, it may be safely said in view of their number, their respectability of character as physi-

---

fever, followed by the characteristic umbilicated eruption which ran regularly through the usual course of vesicle becoming pustule, this filling and forming a crust from its centre, which hardened, dried, and, at the proper time, fell off. Were there any *visible* particle or particles in this 900th in which the specific contagion of variola was contained? Have any such particles of this or of any other contagion ever been found, which will sustain for a moment the idea of its material nature?

cians and men, the unanimity of their testimony and experience, that, in matters of which they have had personal observation, their witness must be accepted as against that of men who, as to matters so testified to, have had none.

Then, are these men combined in efforts to deceive others? In a reply to this question they are entitled to the same candor as other men in relation to other questions. If we accuse a fellow-man of base or mean conduct, we are bound to show. he had at least a possible motive to this, if we ourselves would escape the place of the slanderer. It is confidently asserted, no possible motive can be shown for such a conspiracy; and, that if such a conspiracy were organized, and should succeed in its base object, no good could come to any conspirator, or to any other man, or interest. These witnesses with few, if indeed there be, exceptions, have come to the use of these higher numbers, and to their confidence in them, and to the witness they bring of their efficacy and their superiority as healing agents, by practical demonstrations of these facts in their cures of sick men and women. Their acceptance of these high numbers, in which the microscope can see no drug matter, and their incorporation of them into their practice, have been forced upon them by visible evidence of their superior healing power, and this often, as in the case of the writer, against the strongest prejudices. Their reward has too often been a harvest of hard names, of which fanatic and insane are mild examples, and yet they continue to bear witness to the truth, because it *is* truth.

It has been said and repeated that these dynamists give no report of the failures of these high numbers to cure those for whom they have been prescribed. Well, what then? In this have they not done just as everybody else has done, who has reported cures of cases? One of these objectors, certainly the meanest and most ignorant of them, has gone so far as to say there are many of these. Now we assert boldly of this man, that he knows just nothing at all of the matter he affirms, and that in this statement he has but acted out his accustomed disregard of truth. It is not true of any practice for the cure of the sick that it never fails to cure. All have their failures; and, the judgment as to the superiority of one method over another is a question of ratio of cures to failures, and not of invariable success. But this numerous body of witnesses testify they have found these high numbers more uniformly successful as curatives than lower numbers, and that, for this reason only, they have adopted them into their practice. Very many of them have had abundant experience of both to enable them to form a judgment, which wisdom can hardly pass with contempt. They cure with these more certainly, speedily and pleasantly, or they are either mistaken or false witnesses. Now, who is most likely to be mistaken in the matter of these cures, the man who has seen and knows; or, he who has not seen, and does not know, but only skulks behind his *non credo?*

